# Identifying hepatic genes regulating the ovine response to gastrointestinal nematodes using RNA-Sequencing

**DOI:** 10.3389/fgene.2023.1111426

**Published:** 2023-02-17

**Authors:** Samantha Dixon, Niel A. Karrow, Emma Borkowski, Aroa Suarez-Vega, Paula I. Menzies, Delma Kennedy, Andrew S. Peregrine, Bonnie A. Mallard, Ángela Cánovas

**Affiliations:** ^1^ Centre for Genetic Improvement of Livestock, Department of Animal Biosciences, University of Guelph, Guelph, ON, Canada; ^2^ Department of Pathobiology, Ontario Veterinary College, University of Guelph, Guelph, ON, Canada; ^3^ Department of Population Medicine, Ontario Veterinary College, University of Guelph, Guelph, ON, Canada; ^4^ Ontario Ministry of Agriculture, Food and Rural Affairs, Guelph, ON, Canada

**Keywords:** gastrointestinal nematode (GIN), RNA sequenc ing, ovine, transcriptomics, liver

## Abstract

Gastrointestinal nematode (GIN) infections are considered the most important disease of grazing sheep and due to increasing anthelmintic resistance, chemical control alone is inadequate. Resistance to Gastrointestinal nematode infection is a heritable trait, and through natural selection many sheep breeds have higher resistance. Studying the transcriptome from GIN*-*exposed and GIN-unexposed sheep using RNA-Sequencing technology can provide measurements of transcript levels associated with the host response to Gastrointestinal nematode infection, and these transcripts may harbor genetic markers that can be used in selective breeding programs to enhance disease resistance. The objective of this study was to compare liver transcriptomes of sheep naturally exposed to Gastrointestinal nematode s, with either high or low parasite burdens, to GIN-unexposed control sheep in order to identify key regulator genes and biological processes associated with Gastrointestinal nematode infection. Differential gene expression analysis revealed no significant differentially expressed genes (DEG) between sheep with a high or low parasite burden (*p*-value ≤0.01; False Discovery Rate (FDR) ≤ 0.05; and Fold-Change (FC) of > ±2). However, when compared to the control group, low parasite burden sheep showed 146 differentially expressed genes (64 upregulated and 82 downregulated in the low parasite burden group relative to the control), and high parasite burden sheep showed 159 differentially expressed genes (57 upregulated and 102 downregulated in the low parasite burden group relative to the control) (*p*-value ≤0.01; FDR ≤0.05; and FC of > ±2). Among these two lists of significant differentially expressed genes, 86 differentially expressed genes (34 upregulated, 52 downregulated in the parasited group relative to the control) were found in common between the two parasite burden groups compared to the control (GIN-unexposed sheep). Functional analysis of these significant 86 differentially expressed genes found upregulated genes involved in immune response and downregulated genes involved in lipid metabolism. Results of this study offer insight into the liver transcriptome during natural Gastrointestinal nematode exposure that helps provide a better understanding of the key regulator genes involved in Gastrointestinal nematode infection in sheep.

## 1 Introduction

Gastrointestinal nematode (GIN) infection is a common cause of significant morbidity and mortality in grazing sheep. Due to increasing anthelmintic resistance, chemical treatment alone is inadequate to control these infections. Understanding the genetics of the host response to GINs will be imperative to developing alternative strategies for sheep producers to control infections, such as genetic selection for animals that are more resistant to GIN infection.

Resistance to GIN infection has been defined as the ability of the host to mount an immune response that limits the establishment of the parasite and/or subsequent development of infection (Sweeney et al., 2016). The innate immune system helps protect the host from GIN by supporting the gastrointestinal tract (GIT) barrier, and by producing factors that elicit a rapid and transient inflammatory response that helps to immediately and efficiently clear GINs. Cells of the innate immune system also play a key role in repairing tissues that are damaged by GIN infection. Over time, the acquired immune response also becomes activated and this helps to provide long-term protection against GIN infection by preventing GIN establishment, increasing adult worm mortality and reducing adult egg production (McManus et al., 2014; Sweeney et al., 2016). However, complete knowledge of the extensive host mechanisms used to control GIN infection, and how these contribute to animal-to-animal variation in resistance to GINs, remains incomplete.

Several traits have been utilized as indicators of genetic resistance to GINS, such as fecal egg count (FEC), FAMACHA score to qualitatively measure anemia associated with *Haemonchus contortus* infection, and host immunoglobulin (Ig) responses to GINs (Hunt et al., 2013; McManus et al., 2014). Genomic technologies have also been explored to identify genes and polymorphisms within genes that have a significant association with resistance to parasite infection (McRae et al., 2014; Benavides et al., 2016). However, because of the complex host response mechanisms to GIN infection, no clear consensus has yet emerged concerning the most effective strategy to determine host resistance (McRae et al., 2016). A powerful tool for studying the host mechanisms involved in GIN resistance is transcriptomics, which provides information about gene expression across the whole transcriptome using high-throughput RNA-Sequencing technology (RNA-Seq) (Cánovas et al., 2010; Wickramasinghe et al., 2014). Transcriptomics is particularly important for capturing the dynamic and tissue-specific events that occur during the course of GIN infection (McRae et al., 2016; Sweeney et al., 2016).

The liver participates in the host acute-phase response (APR), which is triggered by exposure to stressors such as microbial infections (Jakab and Kalabay, 1998; Colditz, 2003). Activation of hepatic immune cells contributes to the secretion of pro-inflammatory cytokines such as interleukin (IL)-1β, IL-6, and tumor necrosis factor (TNF)-α during the APR (Kabaroff et al., 2006). Additionally, hepatocytes increase production of acute-phase proteins (APP), while also synthesizing IL-6 to amplify the APR. The APP contribute to the systemic effects of inflammation, which promote pathogen clearance and help restore homeostasis (Robinson et al., 2016). The hepatic APR also influences nutrient availability and uptake by reducing protein synthesis, decreasing fatty acid synthesis and increasing lipolysis in adipose tissue (Malmezat et al., 1998). This is thought to be due to increased nutrient requirements for the production of leukocytes, APP, and Ig (Cheung and Morris, 1984). Walkden-Brown and Eady proposed that costs of immunity to parasites in sheep were attributed to metabolic costs of the host defense (Walkden-Brown and Eady, 2003); pro-inflammatory cytokines, for example, influence the priorities for nutrient utilization, resulting in decreased efficiency of nutrient utilization for growth. Additionally, Colditz found that changes in ovine appetite, growth, and nitrogen metabolism during GIN infection were associated with the systemic effects of the APR (Colditz, 2003).

Functional genomics analysis to explore gene regulatory networks and metabolic pathways associated with host response to GIN infection in sheep will provide a more solid foundation for the identification of genes and genetic variants associated with resistance. Given the liver’s role in regulating the immune and stress responses, especially the APR, investigation of the hepatic transcriptome in sheep during GIN infection is warranted. Therefore, the objective of this study was to examine and compare the liver transcriptomes of sheep naturally exposed to GIN with GIN-unexposed sheep, in order to identify significant differentially expressed genes (DEG) between i) high parasite burden (HPB) and low parasite burden (LPB) sheep, ii) HPB sheep and GIN-unexposed sheep, and iii) LPB sheep and GIN-unexposed sheep, and to perform a comprehensive functional analysis of the list of significant DEG, including gene ontology analysis, metabolic pathways, and gene network analysis to identify biological processes, molecular functions, and metabolic pathways involved in the host response to GIN.

## 2 Materials and methods

### 2.1 Animal and sample collection

The lambs described herein were part of a larger Rideau X Dorset ovine study involving natural exposure to *Haemonchus contortus* GIN (Borkowski, 2019). Animals were managed under the Canadian Council of Animal Care (CCAC) guidelines on the care and use of farm animals in research, teaching, and testing (CCAC, 2009). All procedures were approved by the University of Guelph Institutional Animal Care and Use Committee.

Thirty-eight male and female lambs, born and housed at the Ponsonby Sheep Research Facility, University of Guelph, Ponsonby, ON, were chosen for the study (Borkowski, 2019). The presence of GIN infection has been monitored in this facility since 1988 and remains negative. The male lambs were castrated after selection to reduce differences related to sex hormones. At approximately 1 year of age (age range 8–12 months), 30 sheep (24 females and 6 males) were transported to a commercial farm in central Ontario with a previous history of disease due to GIN infection, mainly *H. contortus*. These study sheep co-grazed with the commercial flock from April to November to allow for natural exposure to mixed GIN infection. In August, one sheep was euthanized due to illness unrelated to parasitemia. A ration of shelled corn mixed with trace mineral salt was fed in metal troughs on pasture at the rate of approximately 0.23 kg per head per day while the lambs were on pasture, and access to water was provided *ad libitum* until slaughter in November.

Eight GIN-unexposed males remained at the Ponsonby Sheep Research Facility and, although they had free-access to pasture, they were not exposed to GIN at this facility. This was confirmed with FEC and visual inspection of the GIT for presence of GINs at slaughter. These lambs were maintained on a first cut hay diet and allowed *ad libitum* access to water and were given approximately 0.23 kg of 80% whole barley/20% whole corn mix (per feeding) twice a week.

The naturally GIN-exposed (n = 29) and GIN-unexposed sheep (n = 8) were slaughtered at the University of Guelph, Department of Animal Biosciences abattoir after 189 days on pasture. Both lamb groups were transported from the farms to a common holding facility less than 1 km from the abattoir and housed indoors as separate groups with *ad libitum* access to hay and water for 72 h prior to slaughter to allow transport-associated stress to abate. Immediately after slaughter, the GITs were collected; the abomasum, small and large intestines were tied off at each end and stored for further analysis of parasite burden. Adult and juvenile worms were speciated using morphologic features *via* microscopy. At the same time, a sample of approximately 5 g of the center of the right lobe of the liver was removed from each animal with a sterile scalpel. The liver samples were taken to a sterile workstation where they were washed in phosphate buffer solution and immediately frozen in liquid nitrogen for transportation. Samples were then stored in −80°C until RNA was isolated.

Among the naturally GIN-exposed sheep, animals with the highest and lowest parasite burdens were selected for RNA-Seq based on GIN count in the abomasum post-slaughter. Animals chosen for RNA-Seq were: high parasite burden (HPB) sheep (n = 6, average GIN parasite count = 270, SD = 240.4), low parasite burden (LPB) sheep (n = 5, average GIN parasite count = 5, SD = 9.8), and GIN-unexposed sheep (n = 4) with no GINs.

#### 2.1.1 RNA extraction, RNA-Seq library preparation and sequencing

A representative sample of tissue (30 mg) from the center of the right liver lobe was ground using a mortar and pestle with liquid nitrogen and homogenized using a hand-held homogenizer. Total RNA was extracted following the manufacturer’s instructions using Qiagen RNeasy Mini Prep Kit (Qiagen, Calif., United States). The quality and quantity of total RNA was assessed using a Nano drop spectrophotometer (Thermo Fisher Scientific, Mass., United States) and an Agilent 2100 Bioanalyzer (Agilent Technologies, Inc., Calif., United States), respectively. All RNA samples used in this study had an RNA integrity value ≥8.0, indicating very good quality.

The NEBNext Ultra RNA Library Prep kit for Illumina (New England Biolabs, Mass., United States) was used for library preparation with Poly-A selection, according to the manufacturer’s recommendations. Library quality and concentrations were assessed using a Qubit fluorometer (Thermo Fisher Scientific, Mass., United States). Sequencing was completed with an Illumina HiSeq 2000 analyzer that yielded 150 base pair (bp) paired-end reads.

#### 2.1.2 RNA-seq analysis

RNA-Seq analysis was performed using the CLC genomics workbench software, version 12 (CLC Bio, Aarhus, Denmark). As described by Cánovas et al. (Cánovas et al., 2014), quality control analysis, including guanine-cytosine (GC) content, ambiguous base content, Phred score, base coverage, nucleotide contributions and over-represented sequences parameters, was performed on fastq files. All the samples passed the quality control analysis showing same length (150 bp), 100% coverage of all bases, 25% of A, T, G and C nucleotide contributions, 50% GC base content and less than 0.1% over-represented sequences. Sequence reads were aligned to the annotated Oar_v3.1 ovine reference genome (release 95). Counts per gene were transformed using a Log10 transformation and normalized to reads per kilo base per million mapped reads (RPKM) values (Izadnia et al., 2019). Differential gene expression analysis was performed between GIN-unexposed (n = 4) and HPB (n = 6) sheep; and GIN-unexposed (n = 4) and LPB (n = 5) sheep; and HPB (n = 6) and LPB (n = 5) sheep using the CLC genomics workbench software, version 12 (CLC Bio, Aarhus, Denmark). Genes were considered to be significantly differentially expressed among groups when they had a *p*-value ≤0.01, a false discovery rate (FDR) ≤ 0.05, and a fold-change (FC) of > ±2. Associated gene name annotation was performed using the Ensembl biomart tool (http://useast.ensembl.org/index.html). For genes with no associated gene name in Ensembl, cDNA sequences were retrieved from Ensembl using Biomart (http://useast.ensembl.org/index.html), and the National Center for Biotechnology Information (NCBI) to perform a “nucleotide BLAST” analysis on Basic Local Alignment Search Tool (BLAST; https://blast.ncbi.nlm.nih.gov/Blast.cgi) with the following options: against the nucleotide collection, with no specified species, and optimize for highly similar sequences megablast, E-value = ≤0.01, NCBI % alignment = 91%–100%, and query cover = 24%–100% (Supplementary Table S1).

#### 2.1.3 Functional analyses

Gene ontology (GO) enrichment analysis was performed using WEB-Based Gene SeT AnaLysis Toolkit software (http://www.webgestalt.org) using the following parameters: minNum (minimum number of genes per category) = 5 of the total genes in the input, fdrMethod (FDR method) using BH (Benjamini–Hochberg), sigMethod (Significant method) using FDR, and fdrThr (FDR threshold) of ≤0.05. The GO terms associated with the three main GO categories (biological process, molecular function, and cellular component) were analyzed (Wang et al., 2013). These analyses were performed on the upregulated and downregulated list of the 86 significant DEG that were found among the LPB *versus* GIN-unexposed and HPB *versus* GIN-unexposed groups (*p*-value ≤0.01, FDR ≤0.05, and FC of > ±2).

Metabolic pathway analyses and regulator effect analyses were performed on the same list of 86 significant DEG common among the LPB and HPB *versus* unexposed groups using Ingenuity Pathway Analysis (IPA) (Ingenuity Systems, Inc., http://www.ingenuity.com). Thresholds of *p*-value ≤0.01, FDR ≤0.05, and FC > ±2 were applied to filter significant genes for function, enriched metabolic pathway, and gene network analyses effects.

## 3 Results

### 3.1 Sequencing and alignment-basic statistics

Each of the 15 RNA-Seq samples generated an average 56,726,456 paired-end reads per sample (SD = 5,260,581.8), with an average input read length of 150 bp. RNA-Seq analysis revealed an average 82.09% (SD = 4.5) of the reads were mapped to the annotated Oar_v3.1 ovine reference genome (release 95). The alignment rates were similar to others using ovine liver, with mapped reads averaging ∼71%–∼83% (Alvarez Rojas et al., 2015; Sabino et al., 2018; Izadnia et al., 2019).

### 3.2 Differentially expressed genes between exposed and unexposed sheep

**FIGURE 1:**
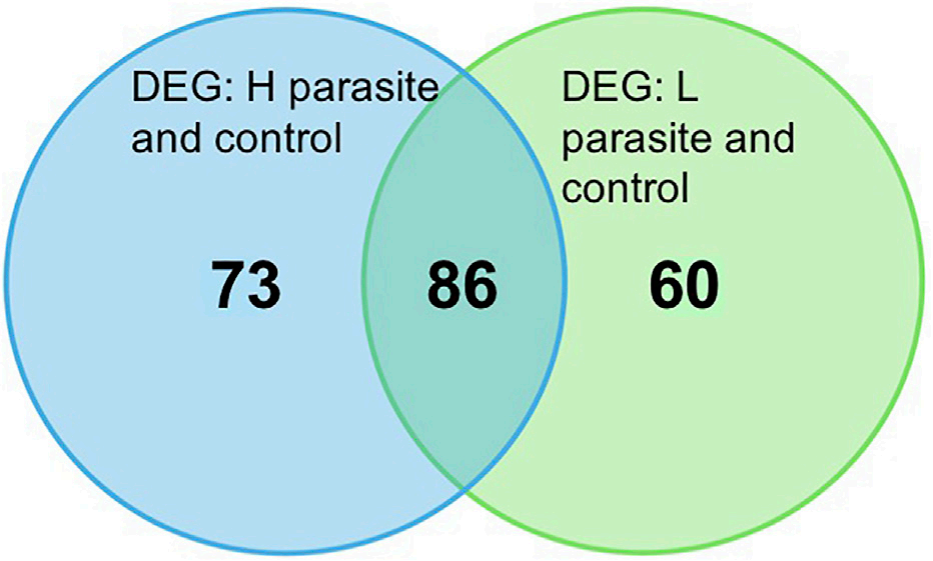
Venn diagram of number of significant differentially expressed genes (DEG; *p*-value ≤0.01, FDR ≤0.05, and FC > ±2) between high parasite burden (HPB) sheep *versus* GIN-unexposed group, and low parasite burden (LPB) sheep *versus* GIN-unexposed group.

No significant DEG were found between the HPB and LPB groups; however, when comparing the list of DEG between HPB and LPB groups and GIN-unexposed group, 86 significant DEG were common to both parasite burden groups (34 upregulated and 52 downregulated in the parasited group relative to the control) (*p*-value ≤0.01, FDR ≤0.05, and FC > ±2). This list of DEG represents a gene profile of GIN-exposed animals compared to GIN-unexposed animals, regardless of parasite burden, and will be referred to as the DEG in the GIN-exposed group for the remainder of this paper. There were also 60 genes significantly differentially expressed (*p*-value ≤0.01, FDR ≤0.05, and FC > ±2) found only in the LPB group when compared to GIN-unexposed group (30 downregulated, 30 upregulated in the LPB group relative to the control), and 73 genes significantly differentially expressed (*p*-value ≤0.01, FDR ≤0.05, and FC > ±2) found only in the HPB group compared to GIN-unexposed group (50 downregulated, 23 upregulated in the HPB group relative to the control) (Figure 1). Supplementary Table S2 highlights the ten most highly expressed genes (based on RPKM values), and ten most highly upregulated and downregulated genes (based on FC values) found among the DEG in the GIN-exposed group.

The most upregulated DEG compared to the GIN-unexposed group was *Fibroblast Growth Factor 21* (*FGF21*), with FC = 28.72 in the HPB (*p*-value = 5.61E-17, RPKM = 67.69, FDR = 3.68E-13) and FC = 17.2 in the LPB groups (*p*-value = 5.37E-07, RPKM = 36.08, FDR = 2.11E-04). In contrast, the most downregulated DEG was *Thyroid Hormone Responsive* (*THRSP*), with FC = −61.9 in the LPB (*p*-value = 6.04E-53, RPKM = 2.37, FDR = 1.58E-48); and FC = −28.9 in the HPB groups (*p*-value = 2.97E-36, RPKM = 5.59, FDR = 3.90E-32).

Among the significant DEG in the GIN-exposed group (*p*-value ≤0.01, FDR ≤0.05, and FC > ±2), the most highly expressed gene, based on the RPKM value, was *beta-2-microglobulin* (*B2M*) with an RPKM value of 1904.48 in the LPB (*p*-value = 8.4E-08, FC = 2.25, FDR = 4.04E-05) and 1948.48 in the HPB groups (*p*-value = 2.0E-05, FC = 2.09, FDR = 4.20E-03).

Two other highly expressed DEG associated with host response to GINs were also directly related to the major histocompatibility complex (MHC); *Ovis aries OLA class I histocompatibility antigen, alpha chain BL3-7-like I* (*OLA-I, ENSOARG00000015002*) with an RPKM value of 1111.82 in the LPB (*p*-value = 3.6E-08, FC = 2.77, FDR = 1.99E-05) and 1524.85 in the HPB groups (*p*-value = 7.1E-08, FC = 3.36, FDR = 4.16E-05), and *CD 74 Molecule* (*CD74*) with an RPKM value of 244.65 in the HPB (*p*-value = 3.5E-06, FC = 2.38, FDR = 9.75E-04) and 213.55 in the LPB groups (*p*-value = 1.1E-12, FC = 2.34, FDR = 2.22E-09).

Another highly expressed gene in the GIN-exposed group was *alpha-1-acid glycoprotein* (*ORM1*), with RPKM values of 483.04 in the LPB (*p*-value = 2.3E-13, FC = 10.51, FDR = 6.13E-10) and 769.54 in the HPB groups (*p*-value = 2.0E-06, FC = 15.20, FDR = 6.00E-04). Similarly, the *Haptoglobin* (*HP*) gene was also highly expressed, with RPKM values of 2496.80 in the LPB (*p*-value = 7.7E-09, FC = 18.23, FDR = 5.16E-06) and 959.43 in the HPB groups (*p*-value = 1.2E-05, FC = 6.44, FDR = 2.68E-03).

Along with being one of the most highly expressed genes in both GIN-exposed groups, *ORM1* was also the second most upregulated gene, with FC = 15.20 in the HPB and FC = 10.51 in the LPB groups. Similarly, *HP* was also highly upregulated with FC = 18.23 in LPB and FC = 6.44 in HPB groups.

The *CAMP Responsive Element Binding Protein 3 Like 3* (*CREB3L3*) gene was also commonly upregulated in the GIN-exposed group, with an RPKM value of 256.72 in the HPB (*p*-value = 3.6E-06, FC = 2.84, FDR = 9.94E-04) and 192.96 in the LPB groups (*p*-value = 1.1E-04, FC = 2.33, FDR = 1.92E-02).

### 3.3 Functional analysis of DEG between GIN-exposed and unexposed groups

The GO terms associated with the three main GO categories (biological process (BP), molecular function (MF), and cellular component (CC)) were analyzed. The 52 common significant downregulated genes between GIN-exposed and unexposed groups were clustered in 21 GO terms (11 in BP, 5 in MF, and 5 in CC categories (Supplementary Table S3). The enriched terms in the BP-GO category were related to metabolic processes, with the most significant being “*small molecule metabolic process*” (*p*-value = 2.68E-36, FDR = 6.32E-17). The “*cofactor binding*” (*p*-value = 1.11E-16, FDR = 3.93E-13) was the most enriched GO term in the MF category and the “*endoplasmic reticulum membrane*” (*p*-value = 4.00E-11, FDR = 3.25E-08) was the most enriched in CC category. The 34 common upregulated genes were clustered in 18 GO terms (10 in BP and 8 in CC) (Supplementary Table S4). Among the BP terms, all were related to the immune response, with “*acute inflammatory response*” being the most enriched (*p*-value = 3.81E-09, FDR = 3.47E-05). Related to the immune system, “*MHC protein complex*” (*p*-value = 3.81E-06, FDR = 0.01) was also the most significant CC term.

Downregulation of metabolic processes and upregulation immune response pathways were also seen in the functional network and metabolic pathway analysis using IPA software. The top significant canonical metabolic pathways identified (*p*-value ≤0.01) were *cholesterol biosynthesis*, along with the top molecular and cellular functions such as *lipid metabolism, small molecule biochemistry, vitamin and mineral metabolism, molecular transport* and *amino acid metabolism* (Supplementary Table S5).

**FIGURE 2:**
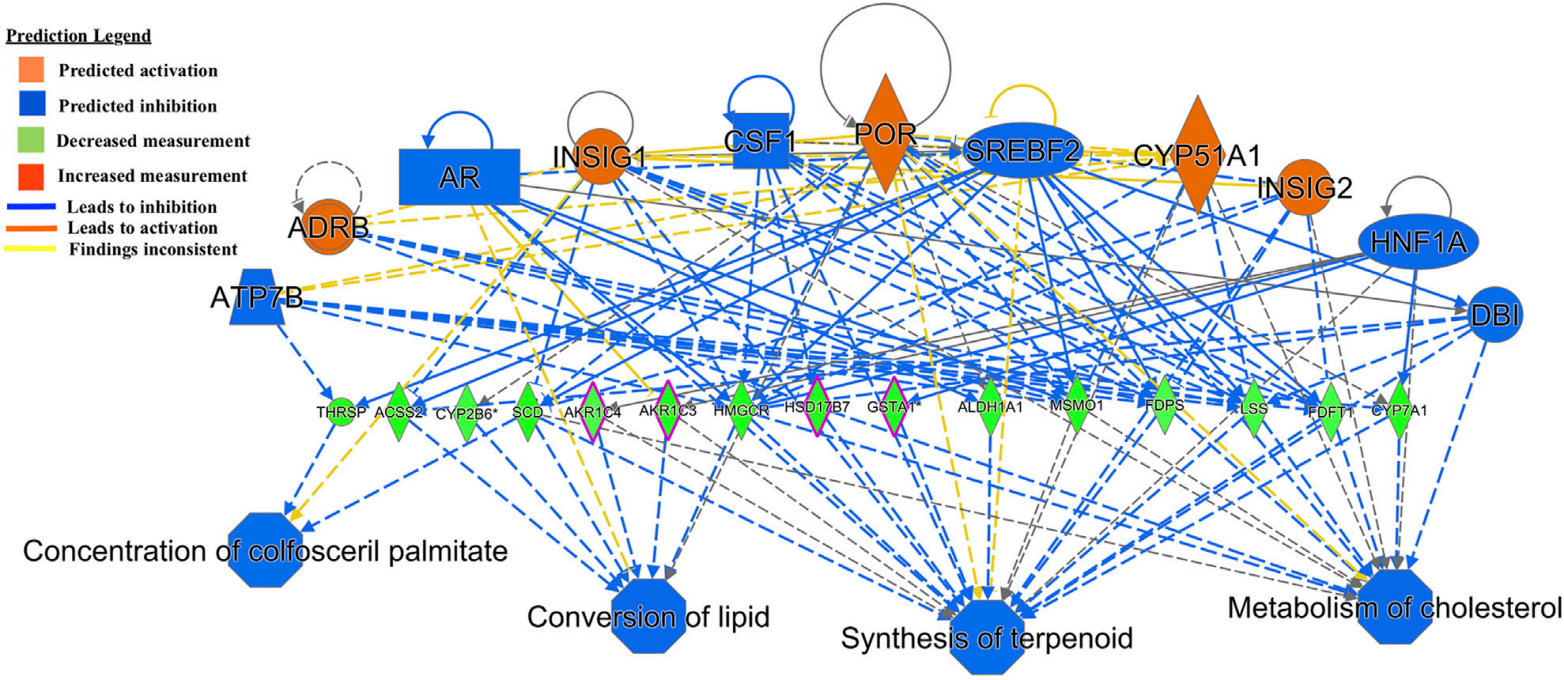
Functional network and metabolic pathway analysis showing inhibition of metabolic pathways from the list of 86 significant differentially expressed genes in the GIN-exposed *versus* GIN-unexposed group (*p*-value ≤0.01, FDR ≤0.05, and FC > ±2).

The regulator effect function in IPA connects upstream regulators, dataset genes and downstream functions or diseases. In the GIN-exposed group, the first regulator effect network (Figure 2) had notable regulators of *Insulin-induced Gene* (*INSIG1/2*) and *Cytochrome P450 Family 51 Subfamily A Member 1* (*CYP51A1*). Both these genes affect multiple genes that were all downregulated, such as *Stearoyl-CoA Desaturase* (*SCD*) and *THRSP,* which were the most highly downregulated genes among the GIN-exposed group*.* These genes lead to the predicted inhibition of functions such as *metabolism of cholesterol, synthesis of terpenoids, conversion of lipid,* and *concentration of colfosceril palmitate*.

**FIGURE 3:**
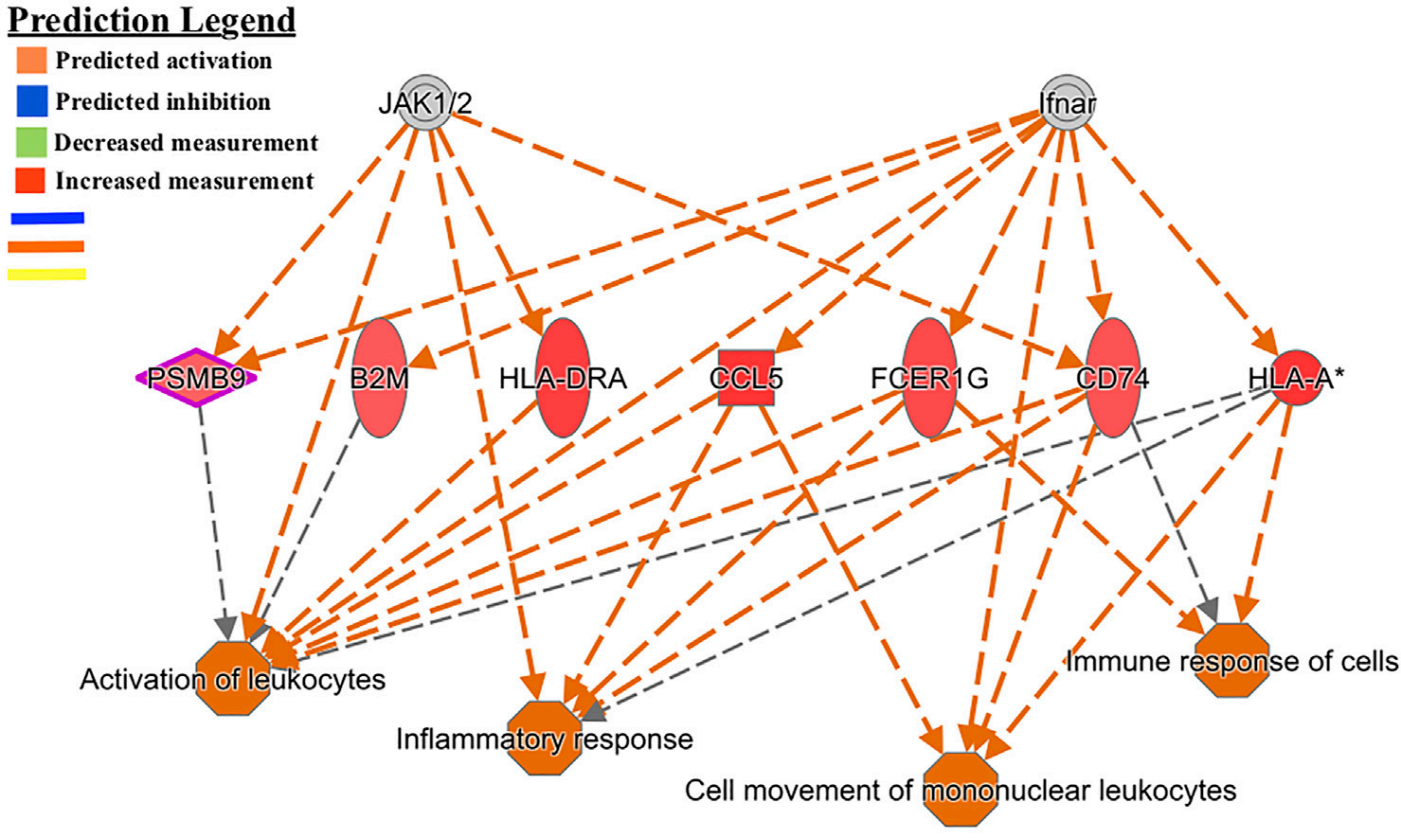
Functional network and metabolic pathway analysis showing activation of immune response pathways from the list of 86 differentially expressed genes in the GIN-exposed *versus* GIN-unexposed group (*p*-value ≤0.01, FDR ≤0.05, and FC > ±2).

**FIGURE 4:**
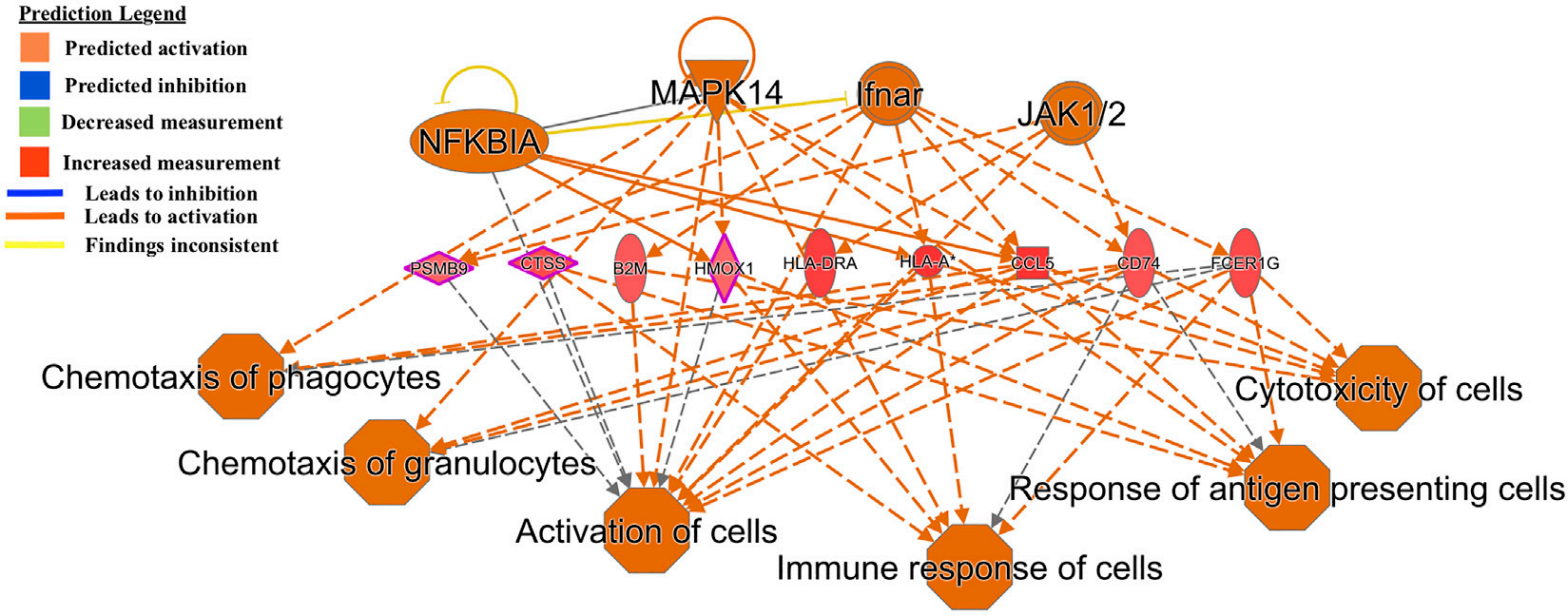
Functional network and metabolic pathway analysis showing activation of immune response pathways from the list of 86 differentially expressed genes in the GIN-exposed *versus* GIN-unexposed group (*p*-value ≤0.01, FDR ≤0.05, and FC > ±2).

Another regulator-effector network led to the predicted activation of functions such as *activation of leukocytes, inflammatory response, cell movement of mononuclear leukocytes,* and *immune response of cells* (Figure 3). A similar effector network led to the predicted activation of immune functions such as *chemotaxis of phagocytes and granulocytes, immune response of cells, response of antigen presenting cells,* and *cytotoxicity of cells* (Figure 4).

## 4 Discussion

### 4.1 Differentially expressed genes between exposed and unexposed sheep

The most highly expressed gene among the significant DEG in the GIN-exposed sheep, in both LPB and HPB groups, when compared to controls, was *B2M*. The *B2M* gene encodes a protein that associates with the class I MHC alpha chain, which is involved in presenting endogenous antigens to cytotoxic CD8^+^ T-cell (Liu et al., 2018). This is an important pathogen-recognition mechanism that ensures a host immune response to potential internal threats (Venturina et al., 2013). The *B2M* gene has been found to be highly expressed in GIN-resistant sheep in several studies (Keane et al., 2007; Ahmed et al., 2015; Berton et al., 2017); these complements the finding in the present study and validate the importance of this gene during GIN exposure, as it was the most highly expressed gene in the GIN-exposed group.

The most upregulated DEG compared to the GIN-unexposed group was *FGF21*. This protein is a secreted endocrine factor that functions as a major metabolic regulator. Its expression is altered by physiological, metabolic and environmental factors (Erickson and Moreau, 2016). Circulating levels of FGF21 are increased in response to fasting and environmental stress (Schaap et al., 2013). Because this gene is altered in response to many different factors, it cannot be concluded that the GIN infection has upregulated the expression of this gene in the GIN-exposed groups; factors such as the different environments and slightly different diets between the GIN-unexposed and GIN-exposed groups could also be influencing its expression. Non-etheless, there is a connection between the immune and endocrine systems *via* the endocrine-immune axis (Dhabhar and McEwen, 1997).

The most downregulated DEG was *THRSP*. The protein encoded by this gene is involved in lipid metabolism, specifically, cholesterol synthesis. Lipid metabolism is regulated during the host response to infection; however, parasites also induce significant changes in lipid metabolism (Grunfeld and Feingold, 1996). Candidate mechanisms for such changes include altered gastrointestinal hormones and epithelial function, and changes in the number and type of immune cells in metabolic tissues (Bansal et al., 2005). This gene, being the most downregulated in the GIN-exposed group compared to the unexposed group, may support this evidence that GINs alter host lipid metabolism.

Interestingly, two other highly expressed genes associated with host response to GINs were also directly related to MHC, *OLA-I* and *CD74*; the *OLA-I* gene is associated with MHC I (Liu et al., 2018), whereas, the CD74 protein encoded by *CD74* associates with MHC class II and regulates exogenous antigen presentation during the immune response (Schröder, 2016). Alleles OMHC1-188 and OLADRB2-282 of the ovine MHC have previously been associated with GIN resistance (Outteridge et al., 1996; Figueroa Castillo et al., 2011), and quantitative trait loci (QTL) studies of the ovine MHC region on chromosome 20, have found a statistically significant association with GIN resistance (Outteridge et al., 1996; Keane et al., 2007; Stear et al., 2007). Our study reaffirms the importance of ovine MHC during GIN exposure, as the most highly expressed hepatic genes in the GIN-exposed group were associated with MHC.

Another highly expressed gene in the GIN-exposed group was *ORM1*. This gene encodes for the orosomucoid APP, and its expression is regulated by glucocorticoids, IL-1, TNF-α, and IL-6 (Luo et al., 2015). The *ORM1* gene is an immunomodulator that is induced by stressful conditions, such as GIN infections. Receptors for ORM1 have been found on macrophages and neutrophils, and activation has been shown to suppress pro-inflammatory gene expression (Lee et al., 2010). Orosomucoid also aids in B- and T-cell maturation (Okumura et al., 1985; Fournier et al., 2000) and has been shown to participate in the innate immune response by binding directly with bacterial lipopolysaccharide and neutralizing its toxicity (48).

The *HP* gene was another highly expressed gene in the GIN-exposed group. Haptoglobin is another important APP, functioning to both inhibit mast cell proliferation and suppress T-cell proliferation, as part of its integral immunomodulatory and anti-inflammatory role, and also to sequester iron (Murata et al., 2004). Along with being one of the most highly expressed genes in both GIN-exposed groups, *ORM1* was also the second most upregulated gene. Similarly, *HP* was also highly upregulated. This further contributes to the findings that the APR may be an important part of the host response to GIN and warrants further investigation.

The *CREB3L3* gene was also commonly upregulated in the GIN-exposed group; *CREB3L3* is associated with acute inflammatory responses and hepcidin expression (Canali et al., 2016). Hepcidin values increase in response to inflammatory cytokines and low iron levels (Abreu et al., 2018), and thus may be involved during *H. contortus* infection.

### 4.2 Functional analysis of DEG between GIN-exposed and unexposed groups

Associated GO terms and the functional network and metabolic pathway analysis of the downregulated genes between GIN-exposed and unexposed groups were related to metabolic processes, whereas, the upregulated genes were all were related to the immune response.

Common genes that lead to immune activation in two of the functional networks (Figures 3, 4) were *CD74*, *B2M* and *OLA-I*. These genes were significantly highly expressed among the GIN-exposed group. These immune functions are necessary for controlling GIN infection; for example, *chemotaxis of granulocytes* involve neutrophils, which are part of the innate response to GINs, and *antigen presentation*, involving the MHC complex, is required for T-cell to respond to GINs (Alba-Hurtado and Muñoz-Guzmán, 2013; Liu et al., 2018).

### 4.3 Limitations of the study

How GIN-exposed and GIN-unexposed sheep were managed during natural challenge could be a limiting factor in the present study. In order to ensure pastures were devoid of GINs, the GIN-unexposed group of sheep were maintained at the Ponsonby Sheep Research Facility for the grazing season. This biosecure facility is free of GIN parasites and is routinely monitored using FEC. Although these animals had access to pasture, the main diet was hay, unlike the GIN-exposed animals, which were fed a primarily pasture-based diet. Both groups were fed a grain mix of similar composition. It is possible that this difference in diet may have affected the liver transcriptome, more specifically metabolic pathways, as it is one of the main metabolic processing organs. However, since the immune response DEG appeared to be related to GIN exposure, these genes are more likely to be differentially expressed due to the exposure of GINs.

Another important consideration of the present study is that liver samples were collected at the end of grazing season (early November) when adult GIN burdens are typically lower and recently ingested L3 are likely becoming hypobiotic. The host immune response to GINs is most active during and shortly after the peak infection time (Mederos et al., 2010); FECs in the GIN-exposed sheep confirmed peak infection occurred during August (CCAC, 2009). Thus, the transcriptomic profile of the liver samples in this study provided insight into the transcriptome during the resolution phase of GIN infection, as opposed to during peak GIN infection.

Since the liver receives 80% of its blood supply from the hepatic portal vein after circulating through the GIT (Robinson et al., 2016), transcriptomic analysis of this tissue provides indirect evidence of GIN infection as the immune cell population of the liver will be actively surveying for pathogens coming from the GIT. However, the liver is also where the APR is initiated and where APP are produced (Colditz, 2003), providing insight into the role of the APR during GIN exposure. Therefore, although the liver transcriptome does not provide a gene profile directly associated with GIN infection, unlike tissues where GINs attach and feed off the host such as the abomasum, it does provide a more systemic and indirect view of the host response to GIN infection.

## 5 Conclusion

The sheep exposed to GINs, regardless of parasite burden, showed an overall decrease in genes associated with metabolic processes and an increase in genes associated with immune processes, compared to the GIN-unexposed group. These results highlight that the host immune response to GIN infection remains activated after peak infection, while the GIN may influence metabolic genes making metabolic processes less efficient. Using the results from this study, the key regulator genes found in relation to immune response (*B2M, CD74, OLA-I, HP* and *ORM1*) and lipid metabolism (*FGF21* and *THRSP*) should be further explored to identify functional structural variants (i.e. single nucleotide polymorphisms, insertions and deletions, and splice variants) that may contribute to their variation in response to GIN resistance. Such work could potentially contribute to the possible development of genetic tests for the resistant trait to GIN infection, and/or their implementation as potential functional genetic markers in breeding programs to enhance GIN-resistance in sheep.

## Data Availability

The original contributions presented in the study are publicly available. This data can be found here: https://www.ncbi.nlm.nih.gov/geo. Accession Number: GSE224936.
